# Niche Suitability Affects Development: Skull Asymmetry Increases in Less Suitable Areas

**DOI:** 10.1371/journal.pone.0122412

**Published:** 2015-04-15

**Authors:** Renan Maestri, Rodrigo Fornel, Daniel Galiano, Thales R. O. de Freitas

**Affiliations:** 1 Programa de Pós-Graduação em Ecologia, Universidade Federal do Rio Grande do Sul, Porto Alegre, RS, Brazil; 2 Programa de Pós-Graduação em Ecologia, Universidade Regional Integrada do Alto Uruguai e das Missões, Erechim, RS, Brazil; 3 Programa de Pós-Graduação em Biologia Animal, Universidade Federal do Rio Grande do Sul, Porto Alegre, RS, Brazil; 4 Departamento de Genética, Universidade Federal do Rio Grande do Sul, Porto Alegre, RS, Brazil; Consiglio Nazionale delle Ricerche (CNR), ITALY

## Abstract

For conservation purposes, it is important to take into account the suitability of a species to particular habitats; this information may predict the long-term survival of a species. In this sense, morphological measures of developmental stress, such as fluctuating asymmetry, can be proxies for an individual’s performance in different regions. In this study, we conducted tests to determine whether areas with different levels of suitability for a species (generated by ecological niche models) were congruent with morphological markers that reflect environmental stress and morphological variance. We generated a Maxent niche model and compared the suitability assessments of several areas with the skull morphology data (fluctuating asymmetry and morphological disparity) of populations of the Atlantic forest endemic to Brazil rodent *Akodon cursor*. Our analyses showed a significant negative relationship between suitability levels and fluctuating asymmetry levels, which indicates that in less suitable areas, the individuals experience numerous disturbances during skull ontogeny. We have not found an association between morphological variance and environmental suitability. As expected, these results suggest that in environments with a lower suitability, developmental stress is increased. Such information is helpful in the understanding of the species evolution and in the selection of priority areas for the conservation of species.

## Introduction

Conservation strategies, such as the choice of priority areas for conservation, take into account the characteristics of the habitat fragment (size, shape, sustained biodiversity) [1], human impacts associated with political and social interactions [2,3], and potential for restoration [4,5]. Occasionally, a flagship species is used for its charismatic appeal to attract more attention from the authorities and the general public to the area to be conserved [6,7]. However, whether the individuals of a species in a particular area are well or poorly adapted to the environment in which they live is rarely considered. Due to the results of studies using the optimum niche theory, which states that a particular combination of condition and resources allow the species to maintain a viable and reproductive successfully population [8], it is known that some regions are more suitable for a species than other areas, based on factors such as the climatic conditions, availability of resources, and abundance of predators [9,10]. These conditions are related to the evolution of a species in certain habitats and are directly related to the long-term survival of the species, which is of fundamental importance to the long-term maintenance of biodiversity in protected areas.

In this context, ecological niche modeling is a useful tool because of its ability to find highly suitable habitats for the focus species, as noted in many conservation studies [11–14]. Identifying areas with the appropriate environmental conditions to sustain a species is fundamental to conservation studies [15]. Moreover, niche modeling techniques can be useful for quantifying the limits of the fundamental niche of species and for predicting current and potential species distribution across landscape [16,17]. Nevertheless, it is worth remembering that the output information of the models does not take into consideration any measurement of the performance of the individuals of a species in different areas. Therefore, the relationship between optimum niche theory and models of species distribution requires a more integrative approach [18–20].

Fluctuating asymmetry (FA) is an accepted measure of individual capability that can be directly related to developmental stability [21–26], but see [27] for a review of our knowledge about the underlying process of developmental noise, and a criticism about the general applicability of FA as a measure of fitness. FA can be characterized as the difference between the right and left sides of symmetric parts (such as arms, i.e., matching symmetry) or between bilateral parts of symmetric organisms (such as the right-side and left-side of a skull, i.e., object symmetry) [28,29]. Thus, FA is a measure of genetic and possible environmental stress during development; organisms that are more symmetrical are also more successful in stabilizing structures during development [30], and the opposite phenomenon is also true. Therefore, FA is a proxy for ontogenetic developmental disturbances [31]. Thus, increased asymmetry tends to occur in stressed marginal habitats, which are commonly found in regions outside the environmental optimum of a species [32].

Herein, for the first time, we combine the two approaches (fluctuating asymmetry and niche modeling) to determine whether the patterns of fluctuating asymmetry and morphological variance indicate highly suitable areas. Our objective was to test for a correlation between areas with different levels of environmental suitability and different levels of FA and variability in morphology. Our initial hypothesis was that the lower was the average fluctuating asymmetry of a population (more symmetric individuals) more suitable would be the area that this population inhabits. We expected a negative relationship between fluctuating asymmetry and suitability, based on the fluctuating asymmetry theory [26,29,31]. In contrast, we expected a positive correlation between morphological variance and suitability. The latter hypothesis was based on previous findings that have shown that patterns of genetic variability (nucleotide and haplotype diversity) correlate positively with highly suitable areas [14]. Other studies have also shown interactions between genetic markers and niche modeling [33,34], with implications for conservation. Also, environmental and geographical variables were shown to be related with skull shape and size of mammals in several ways [35,36,37,38]. For instance, the longitudinal cline of skull morphological variance in *Cercopithecus* monkeys, and its relationship with rainfall [35], and the morphological difference related with altitudinal difference in the rodent *Akodon mollis* [36]. In this study, we applied morphological markers with niche models, and we expected that the information generated would potentially to lead to new conservation strategies. The approach we propose takes into account the adaptability of species to particular environments when assessing priority areas for biodiversity conservation.

To test these hypotheses, our study focused on the species *Akodon cursor*. A rodent endemic to the Brazilian Atlantic forest, *Akodon cursor* is a small-bodied species (around 50g) [39], which has an extended geographical distribution, occurring from 5° to 25° south latitude in localities ranging from sea level to 1,170 m, and is the most abundant Sigmodontine rodent of the biome [39]. The species is classified as LC (Least Concern) by the IUCN Red List [40]. Despite this, the Brazilian Atlantic forest is a priority area for worldwide biodiversity conservation because of its great diversity and the level of threat that it faces [1,41], which is corroborated by the fact that only approximately 12% of its original vegetation remains, and the majority of the remaining vegetation covers fewer than 50 hectares [1].

## Materials and Methods

### Species distribution modeling

The species distribution modeling (SDM) for *A*. *cursor* occurrence in the Atlantic forest biome was generated by the maximum entropy algorithm implemented in Maxent software 3.3.3e [42,43]. Maxent is a machine learning program that estimates the probability distribution for a species occurrence based on environmental constraints [42]. Maxent estimates the ecological niche of a species by determining the distribution of maximum entropy (ME) subject to the constraint that the expected value of each environmental variable under this estimated distribution matches its empirical average. Maxent output provides a species’ distribution model of environmental suitability in the geographic space, which ranges from 0 (unsuitable) to 1 (highly suitable). These areas of high suitability can be understood as areas where the environmental conditions meet the niche requirements of a species [42,43]. We chose Maxent because it only requires presence data, because it can process categorical data and model interactions, and because it has performed favorably when compared to alternative approaches [44,45]. Different studies have demonstrated the utility of species distribution modeling for identifying areas of high conservation value using Maxent [11] or ensemble models [46], with Maxent showing, in general, the best performance [46–49].

**Fig 1:**
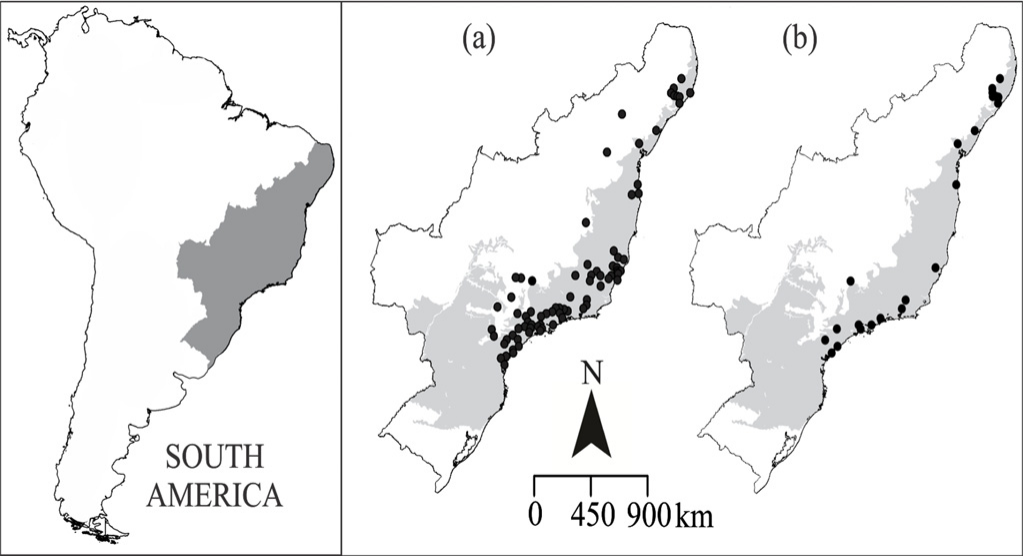
Geographic distribution of sampling localities of *Akodon cursor*. A) 84 presence data entries used in the species distribution modeling. B) 22 localities used for the fluctuating asymmetry and morphological variance analysis. Light gray represents the boundaries of the Atlantic forest biome. South America map obtained from OpenStreetMap (free available at: http://www.openstreetmap.org/), and Atlantic forest biome shape obtained from MMA (free available at: http://mapas.mma.gov.br/i3geo/datadownload.htm). The image was edited using CorelDraw graphics Suite.

Models were generated using presence-only data (N = 80) (S1 Appendix, Fig. 1) and environmental layers at a spatial resolution of 0.0083 decimal degree (~1 km^2^). We used 19 WorldClim bioclimatic layers obtained with interpolated data derived from rainfall and temperature, plus one elevation and one land cover layer (land cover map, available at http://due.esrin.esa.int/globcover/). All the layers were converted to rasters at the same level of resolution as the bioclimatic layers using ArcMap v. 10.0 and clipped for the Atlantic Forest biome boundaries. We generated a matrix with the values of each climatic layer for the entire study area. We performed a principal components analysis (PCA) on this matrix to identify correlations between variables, selecting the axes that explain 95% of the correlation structure. From this result, we selected the layers with the highest absolute coefficient in each axis. This procedure yielded five layers for *A*. *cursor* (Table 1).

**Table 1:** Environmental variables used for species distribution modeling (SDM) for *Akodon cursor* in the Atlantic Forest biome, Brazil.

Variables	Dataset name	Spatial resolution	Year	Source
Land cover	GlobCover Land; Cover version v2.3	300 meters	2009	ESA GlobCover Project
Elevation	Global elevation data	30 arc-second	2004	NASA Shuttle Radar Topography
Bioclimatic variables	Bio4 = Temperature sazonality; Bio12 = Annual precipitation; Bio19 = Precipitation of coldest quarter	30 arc-second	2005	WorldClim global climatic layers

All presence records were obtained from the SpeciesLink database (http://splink.cria.org.br/) and the registers of specimens at the Museu Nacional do Rio de Janeiro, RJ, Brazil, and the Museu de Zoologia da Universidade de São Paulo, SP, Brazil. All runs were set with a convergence threshold of 1.0^E–5^ with 500 interactions and with 10,000 background points, and the analysis of variable importance was measured by jack-knife, response curves and random seed.

The SDM was generated by bootstrapping methods, with replacements using 70% of the dataset for training and 30% for testing models [50]. We produced maps of the potential distribution of the species using the logistic output format [43]. This format was used in an attempt to ensure the closest possible approach to an estimate of the probability that the species is present in a given environment [51]. The models were validated by calculating the area under the curve (AUC) from a receiver operating characteristic curve (ROC).

### Geometric morphometrics procedures

Digital photographs of the ventral view of the skull of *Akodon cursor* specimens were taken in the mammal collections of the Museu Nacional da Universidade Federal do Rio de Janeiro (MN), Rio de Janeiro, Brazil, and the Museu de Zoologia da Universidade de São Paulo (MZUSP), São Paulo, Brazil. The geographical location of occurrence was recorded for all specimens, and when it was not available in degrees of latitude and longitude, it was recorded as described in notes and then georeferenced. A list of the number of individuals ordered by locality and the list of museum specimens used in this study are available in S2 Appendix. To minimize ontogenetic effects, only adult specimens were photographed. The criterion to separate juveniles from adults was the complete eruption of the third molar.

**Fig 2:**
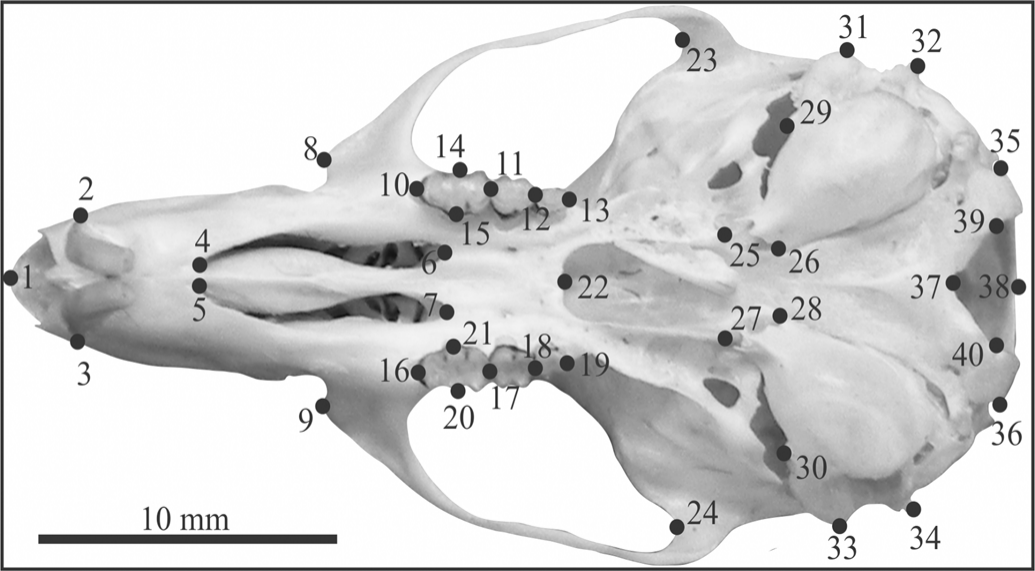
Landmarks digitized in the ventral skull of all *Akodon cursor* specimens. A description of each landmark is presented in S3 Appendix.

Two-dimensional digital images of 380 individuals were taken using a Nikon P100 camera with 13.1 megapixel resolution (3648 x 2736) in the macro function of automatic mode and without flash or zoom. The photos were taken from a standard distance of 50 mm for all specimens. A total of 40 landmarks were digitized in the ventral view of the skull of all individuals on both sides of skull (Fig. 2) using TpsDig2 software [52], and the same person conducted the analysis for each individual (RM). A description of all landmarks is given in S3 Appendix. After digitization, a matrix of the landmark coordinates (x, y) was superimposed using a generalized Procrustes analysis (GPA) in MorphoJ software v1.06b [53]. The GPA removes effects that are not related to shape (position, orientation and scale), creating a new matrix with the shape coordinates only. After superimposition, we checked for outliers in the sample, and these were redigitized to ensure control of the digitizing error. We also tested for the impact of allometry (the association between size and shape) in the entire sample, through a multivariate regression analysis carried in MorphoJ sotware v1.06b [53]. The size of each specimen was accessed through the centroid size: the square root of the sum of squared distances of each landmark from the centroid of the configuration [54]. The average size per population was calculated and used as explanatory variable (see Statistical Analysis section).

We then tested the measurement error based on 50 randomly selected specimens, which were digitized twice in a random order by the same person (RM). The test for the error term was made by a Procrustes ANOVA procedure [55]. The Procrustes ANOVA is an extension of the one-way ANOVA for landmark data, which adds up sums of squares and mean squares over the coordinates of the landmarks and can quantify the amount of shape variation as a measure of the magnitude of the effects in the ANOVA [56,57]. This procedure is fundamental to the control of digitizing error in studies with FA, as FA is a subtle biological effect [27,55,58]. The Procrustes ANOVA for measurement error was performed in MorphoJ software v1.06b [53].

### Quantifying asymmetry and morphological variability

The morphometric analyses of the FA and morphological variance were based on 380 specimens of *A*. *cursor* that were distributed in 22 populations covering virtually the entire geographical distribution of the species (Fig. 1, S2 Appendix). The measure of FA was computed for each individual by a procedure that involves the following: (1) a reflection of each of the original configurations of landmarks (each individual) to its mirror image was made, generating a reflected copy of each configuration; (2) using Procrustes fit, we generated an average of the original and mirrored configurations for each specimen, which is a perfectly symmetric configuration; and (3) the asymmetry of shape was computed for each individual as the deviation of the original configuration of landmarks from the symmetric consensus [29,55,56]. These procedures were performed automatically in MorphoJ software v1.06b [53]. Therefore, for each individual, we had a number (in units of Procrustes distance) that expressed the amount of asymmetry, and we summarized the amount of asymmetry by population, making an average of the FA of all individuals within each population. As a measure of morphological variance, we computed the morphological disparity within each population [59] in the *geomorph* package [60] at R software v.3.03 [61]. The metric (i.e. formula) for morphological disparity is the same used for measuring variation (i.e. the variety of individuals within a single population) [59], therefore we refer here to the variance within populations.

### Statistical analysis

For all analyses, our response variables were the vectors of average FA per population and morphological variance (each containing the one value per population), and our explanatory variables were two vectors of suitability (also with one value per population) generated through niche modeling that were used as environmental suitability values ranging from 0 (minimum suitability) to 1 (maximum suitability). The vectors of suitability were extracted with ArcGis software v. 10.0 for two spatial scales: the local scale (first vector, 1 km²) and the regional scale (second vector, 9 km²). The local scale suitability was extracted from the cell in which the population was present. The regional scale corresponds to the average of suitability of the 9 cells surrounding the focal cell (i.e., population cell). The normality of our response and explanatory variables were checked by visual inspection.

We first tested the spatial autocorrelation of our response variables by two global Moran’s I indices [62] performed in R software v.3.03 [61] with package ape [63]. We conducted this test to determine whether our variables were correlated in space, which could generate bias in our analysis by violating the principles of independence between samples [64]. We also checked if could be an influence of sample size (i.e. number of individuals per locality) upon FA and morphological variance; for this, we ran two independent linear regression analyses with sample size per locality against the vectors of FA and morphological variance. We then ran four independent linear regressions, crossing our response variables against our explanatory variables to determine whether the areas of environmental suitability influenced variability in the morphology and FA of individuals within populations. We also conducted linear regressions to evaluate the relationship between size (average per locality), FA and morphological variance. Linear regressions were performed in R software v.3.03 [61]. In all analyses, the level of significance was α<0.05.

## Results

**Fig 3:**
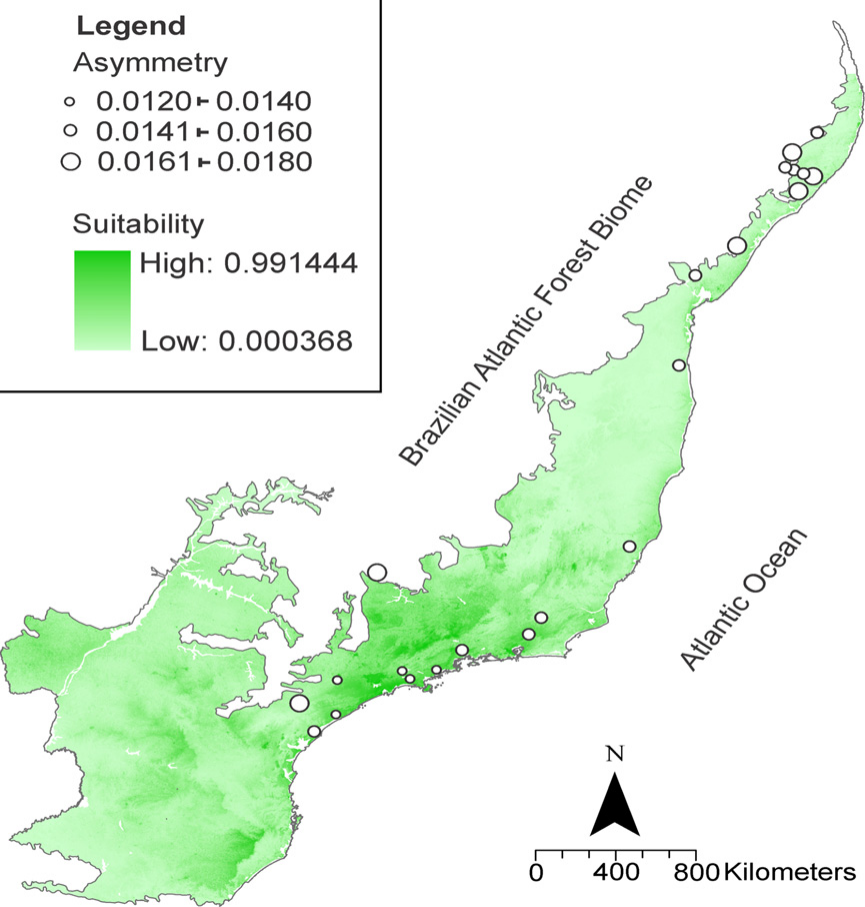
Suitable areas for *Akodon cursor* according to Maxent (maximum entropy) model in Brazilian Atlantic Forest. The white circles indicate levels of fluctuating asymmetry in skulls across geographical space. Atlantic forest biome shape obtained from MMA (free available at: http://mapas.mma.gov.br/i3geo/datadownload.htm), and generated on Quantum Gis (QGIS, free available at: http://qgis.org/).

Based on a maximum entropy modeling algorithm and using five environmental variables, we produced distributional predictions for *A*. *cursor*. Fig. 3 shows the distribution map, with darker colors indicating more suitable habitats and lighter colors indicating unsuitable habitats.

According to the ME model, temperature seasonality (68%) is the variable that most influenced the occurrence of this rodent in the Atlantic forest biome. The other variables have less influence on the distribution of *A*. *cursor* (Land cover, 12.5%; Elevation, 8.6%; Annual precipitation, 6.2%; Precipitation of coldest quarter, 4.7%). For this reason, the amount of temperature variation over a given year appears to be the variable that furnishes the most useful information. Model performance, defined as the area under the curve (AUC), was highly discriminative for the species (AUC = 0.956).

The Procrustes ANOVA indicated that the measurement error is 3.6 times smaller than the individual-by-side interaction (i.e., fluctuating asymmetry) (mean squares for error term: 0.00014678; mean squares for Individual*Side: 0.00053065) and was therefore not a confounding factor in this study. Global Moran’s I showed no spatial autocorrelation in FA (observed: 0.21; expected: -0.047; *P* = 0.062) or in morphological variability (observed: 0.005; expected: -0.09; *P* = 0.52), indicating that the values of FA and morphological variability were randomly distributed in geographical space, which generates no concern about pseudoreplication of the samples. Also, sample size of localities did not influence FA (F_1,20_ = 0.18; *P* = 0.67; R² = 0.009; b = 1^e^05) or morphological variance (F_1,20_ = 1.84; *P* = 0.18; R² = 0.08; b = 5^e^06).

**Fig 4:**
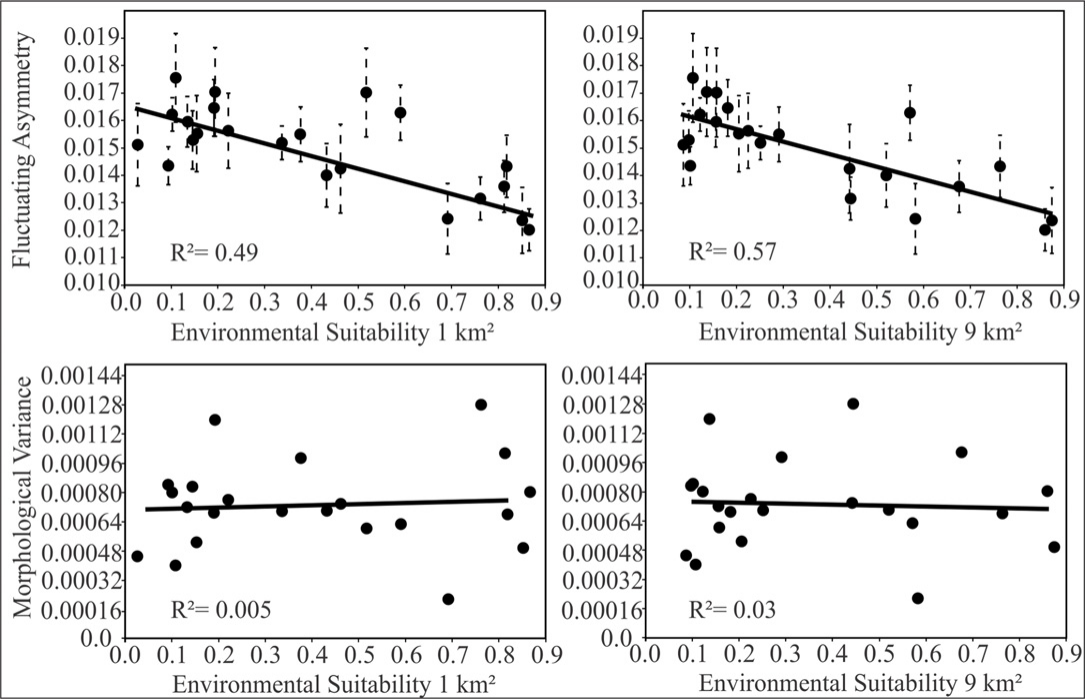
The relationship between fluctuating asymmetry, morphological variability, and environmental suitability. The scatter plots are showing the relationship between fluctuating asymmetry and environmental suitability in two spatial scales (top) and the relationship between morphological variance and environmental suitability (down). The dashed bars represent the standard error. The value of the coefficient of determination is shown.

We found a negative relationship between FA and suitability at the local scale (F_1,20_ = 19.29; *P*< 0.001; R² = 0.491; b = -0.0038) and at the regional scale (F_1,20_ = 27.58; *P*< 0.001; R² = 0.579; b = -0.0046). We found no significant association between morphological variance and suitability at the local scale (F_1,20_ = 0.11; *P* = 0.73; R² = 0.005; b = 6^e^05) or at the regional scale (F_1,20_ = 0.06; *P* = 0.80; R² = 0.003; b = -5^e^05). Scatter plots are shown in Fig. 4. The level of fluctuating asymmetry of the skull of *Akodon cursor* was more pronounced in regions with low environmental suitability and decreased (toward symmetry) as environmental suitability increased. We have not found an association between morphological variability and environmental suitability. Shape variance was 11.92% explained by size (p< 0.001). Nevertheless, we found no relationship between FA and size (F_1,20_ = 2.40; *P* = 0.13; R² = 0.107; b = 6^e^06), neither between morphological variability and size (F_1,20_ = 0.55; *P* = 0.46; R² = 0.026; b = -4^e^07). In Table 2, we presented the Person’s correlation coefficients between FA and all environmental variables, plus the environmental suitability for local and regional scale.

**Table 2:** Pearson’s correlation coefficients (r) between fluctuating asymmetry (FA) and 21 environmental variables, plus environmental suitability in two spatial scales.

Environmental Variables	FA
Annual Mean Temperature	0.481*
Mean Temperature Warmest Quarter	0.331
Mean Temperature Coldest Quarter	0.516*
Annual Precipitation	-0.513*
Precipitation Wettest Month	-0.404
Precipitation Driest Month	-0.386
Precipitation Seasonality	0.208
Precipitation Wettest Quarter	-0.446*
Precipitation Driest Quarter	-0.395
Precipitation Warmest Quarter	-0.634*
Precipitation Coldest Quarter	0.326
Mean Diurnal Range	-0.135
Isothermality	0.585*
Temperature Seasonality	-0.504*
Max. Temperature Warmest Month	0.390
Min. Temperature Coldest Month	0.507*
Temperature Annual Range	-0.417
Mean Temperature Wettest Quarter	0.053
Mean Temperature Driest Quarter	0.590*
Land Cover	-0.087
Elevation	-0.113
Environmental Suitability 1km²	-0.701**
Environmental Suitability 9km²	-0.761**

* indicates statistical significance at *P*<0.05.

** indicates statistical significance at *P*<0.001.

## Discussion

The ME model presented here identified regions that are suitable for *A*. *cursor*. Seasonal temperature variation had the most influence on the distribution of *A*. *cursor*, with temperatures ranging between 20 to 25°C correlating with a higher probability of the presence of the species. The suitable areas for this species in the Atlantic forest biome were mostly restricted to the Coastal Forest on marine sandy soils (Restinga Forest) and interior formations of the biome (Dense Ombrophilous Forest and Deciduous Forest). Despite the fact that the ME model presented areas of medium-high environmental suitability in the southern region of the Atlantic forest (Rio Grande do Sul and Santa Catarina states), the species is not found in this region most likely because other co-generic species such as *A*. *montensis* and *A*. *paranaensis* are very abundant in the southern portion of the biome [65–67], which might impede the presence of *A*. *cursor* due to competition pressure and niche overlap.

Our results allow us to infer that regions with different levels of suitability for *A*. *cursor* were able to influence the adaptability of the individuals because of the specific environmental conditions. Based on the fluctuating asymmetry theory, the higher level of FA in regions with lower suitability is most likely a reflection of individuals’ developmental disturbances in such areas [24,26,29,30,68]. Thus, we have two possible situations. On one hand, this is an indication that the model was successful in identifying differences in suitability in geographical space for this species. Therefore, if we aimed to select areas for conservation units for the preservation of *A*. *cursor*, based on the concordance of the two approaches (FA and SDM), we would be assured that the most suitable areas were really the best possible areas for the long-term viability of the species. On the other hand, if we assume that SDM has good performance in finding regions of high suitability (which was always our premise), then we have identified another useful measure that can provide good evidence for the selection of priority areas for the conservation of a species (i.e., FA). Also, the association between FA and genetic variation could bring new light upon the relationship of the species with its environment, and should be explored.

FA has been used as an indicator of individual capability for a long time [33,69,70], but it is still not widely used for conservation purposes. Here, we suggested that FA might be useful identifying the geographic regions where a species is better adapted to its environment. Despite the fact that FA is not a perfect proxy of environmental stress and fitness because heterogeneous results were found [27], we infer here that FA was able to reflect environmental differences between regions precisely because the FA level is most likely a consequence of the environmental conditions of any area over time. [25] found that macaque skulls that exhibit high levels of asymmetrical deviations also exhibit high levels of environmental variance, showing that environmental variation could be directly related to levels of FA within species. Other studies have noted the relationships among FA and distinct biotic and abiotic interactions. For instance, a high level of FA increases predation risk in mice [71], is related to female preferences in fish [72] and *Drosophila* species [73] and is affected by plantation management for a Carabidae species [74]. All this evidence suggests that FA could be directly related to environmental factors, and the pattern that we found was in fact the outcome of the differences in environmental components among geographic regions, which reflect the suitability of *A*. *cursor* to its environment. In those regions of high suitability, the species most likely inhabits its environmental optimum; in contrast, in the marginal habitats (i.e., low suitability), the species experiences sub-optimal environmental conditions, which leads to developmental disturbances and, consequently, increasing asymmetry [32].

Indirectly, a FA approach takes into account the long-term survival of the species in a given region: regions in which the species experiences its optimal niche will allow the establishment of a healthier population, which will likely have a greater number of individuals compared to those populations in sub-optimal conditions [10]. From a conservation standpoint, the long-term maintenance of biodiversity in protected areas is one of the main goals, and FA may be helpful in this aim. However, there is a very low probability that researchers could obtain a measure of FA in all the species of a certain area or region. Moreover, this measure would have to be taken with the animal alive because it is counterproductive to conservation to sacrifice the animal to take measures of FA. Nevertheless, there are alternative ways to measure FA with the animal alive (e.g., asymmetry in the length of bilateral body parts such as ears, legs, feet), but more studies are necessary to assess how well these alternative measures express FA. On the positive side, FA of museum specimens could help identifying historical process in the evolution of populations at broad geographic scales.

In our study, the regional scale of suitability (9 km²) was better adjusted to FA than the local scale (1 km²), possibly because of random errors that may occur in the local scale due to the resolution of presence records for *A*. *cursor*. Additionally, this may be the result of a more accurate measure of environmental suitability. An entire population occupies more than a local scale, and the average of the cells at the regional scale possibly reflected this broader resolution. Morphological variance, contrary to expectations, was not shown to be correlated with levels of suitability. The variance in morphology could be the result of a wide range of processes [75], for instance, environmental heterogeneity [76], that were not analyzed here.

Our study narrows the boundary between markers of individual capacity (i.e., FA) and conservation by showing that differences in habitat suitability generated by SDM reflect differences in the fluctuating asymmetry within a species. Most likely, the use of indicators of individual capability, such as fluctuating asymmetry, will not be the first priority for conservation managers, at least not with the conservation situation of most faunal species today. Other factors, principally social and economic ones, are currently the most important when a conservation area is selected. However, in an ideal scenario, measures that take in account the adaptability of a species to the environment based on niche theory would be of great help in the selection of priority areas for conservation. This is just another step towards a more comprehensive understanding of the suitability of species to their habitats and of how this may or may not be useful for conservation. Nevertheless, thinking about the future is important, and knowing that we have access to more tools for selecting the best possible areas for maintaining biodiversity must be taken into account. Future studies could measure FA in larger taxonomic samples and observe whether the patterns agree with the results found in our study. Moreover, other indicators of individual capability may be used to more rapidly assess the adaptability of a species in particular areas.

## Supporting Information

S1 Appendix Geographic locality of the presence records of *Akodon cursor* used for species distribution modeling. All records were obtained from the SpeciesLink database and the registers of specimens at the Museu Nacional do Rio de Janeiro, RJ, Brazil, and the Museu de Zoologia da Universidade de São Paulo, SP, Brazil.
(DOCX)

S2 Appendix List of museum specimens. List of museum specimens (N = 380) containing a description of the locality with geographic coordinates in longitude and latitude degrees, the number of individuals by locality, and the museum collection numbers of specimens of *Akodon cursor* used in this study.
(DOCX)

S3 Appendix Description of the 40 landmarks digitized on the ventral skull of the 380 specimens of *Akodon cursor*. (DOCX)
