# Supplementary material for: Niche Suitability Affects Development: Skull Asymmetry Increases in Less Suitable Areas
Source: PLoS One. 2015 Apr 15;10(4):e0122412. doi: 10.1371/journal.pone.0122412 (PMC4398368; doi:10.1371/journal.pone.0122412)
Supplement: S1 Appendix — All records were obtained from the SpeciesLink database and the registers of specimens at the Museu Nacional do Rio de Janeiro, RJ, Brazil, and the Museu de Zoologia da Universidade de São Paulo, SP, Brazil. (DOCX) [file pone.0122412.s001.docx]

**Appendix S1. Geographic locality of the presence records of *Akodon cursor* used for species distribution modeling.** All records were obtained from the SpeciesLink database and the registers of specimens at the Museu Nacional do Rio de Janeiro, RJ, Brazil, and the Museu de Zoologia da Universidade de São Paulo, SP, Brazil.

| **State** | **Municipality** | **Longitude** | **Latitude** |
| --- | --- | --- | --- |
| Alagoas | Capela | -36.081 | -9.411 |
| Alagoas | Quebrângulo | -36.469 | -9.320 |
| Alagoas | São Miguel dos Campos | -36.097 | -9.784 |
| Alagoas | Viçosa | -36.242 | -9.369 |
| Alagoas | Porto de Pedras | -35.322 | -9.161 |
| Bahia | Bonfim | -40.192 | -10.467 |
| Bahia | Feira | -38.975 | -12.268 |
| Bahia | Ilhéus | -39.072 | -14.793 |
| Bahia | Andaraí | -41.261 | -12.802 |
| Bahia | Una | -39.000 | -15.350 |
| Bahia | Camacan | -39.496 | -15.419 |
| Espirito Santo | Santa Teresa | -40.556 | -19.886 |
| Espirito Santo | Santa Leopoldina | -40.533 | -20.100 |
| Espirito Santo | Castelo | -41.092 | -20.518 |
| Espírito Santo | Águia Branca | -40.786 | -18.875 |
| Espírito Santo | Ibitirama | -41.732 | -20.396 |
| Espírito Santo | Viana | -40.461 | -20.359 |
| Espírito Santo | Cariacica | -40.511 | -20.281 |
| Espírito Santo | Domingos Martins | -40.967 | -20.400 |
| Espírito Santo | Itaguaçu | -40.856 | -19.802 |
| Espírito Santo | Governador Lindemberg | -40.480 | -19.260 |
| Espírito Santo | São José do Calçado | -41.719 | -21.043 |
| Espírito Santo | Serra | -40.308 | -20.129 |
| Espírito Santo | Linhares | -40.072 | -19.391 |
| Espírito Santo | Guarapari | -40.505 | -20.667 |
| Minas Gerais | Além Paraíba | -42.697 | -21.876 |
| Minas Gerais | Ouro Preto | -43.522 | -20.389 |
| Minas Gerais | Passos | -46.607 | -20.716 |
| Minas Gerais | Rio Matipó | -42.384 | -20.367 |
| Minas Gerais | Marliéria | -42.650 | -19.717 |
| Minas Gerais | Lima Dutra | -43.883 | -21.700 |
| Minas Gerais | Turmalina | -42.767 | -17.133 |
| Minas Gerais | Passa Quatro | -44.933 | -22.383 |
| Minas Gerais | Fervedouro | -42.483 | -20.717 |
| Minas Gerais | Simonésia | -42.000 | -20.133 |
| Paraná | Guaratuba | -48.581 | -25.894 |
| Paraná | Morretes | -48.840 | -25.480 |
| Paraná | Guaraqueçaba | -48.468 | -25.357 |
| Pernambuco | Bom conselho | -36.685 | -9.172 |
| Pernambuco | Caruaru | -35.955 | -8.277 |
| Pernambuco | Garanhuns | -36.496 | -8.883 |
| Rio de Janeiro | Angra dos Reis | -44.479 | -22.994 |
| Rio de Janeiro | Teresópolis | -42.950 | -22.414 |
| Rio de Janeiro | Itatiaia | -44.670 | -22.390 |
| Rio de Janeiro | Bonsucesso | -42.733 | -22.200 |
| Rio de Janeiro | Volta Redonda | -44.065 | -22.575 |
| Rio de Janeiro | Porto Real | -44.334 | -22.467 |
| São Paulo | Guararema | -46.038 | -23.409 |
| São Paulo | Salto de Pirapora | -47.580 | -23.652 |
| São Paulo | Salesópolis | -45.900 | -23.650 |
| São Paulo | Ubatuba | -45.117 | -23.417 |
| São Paulo | Capão Bonito | -48.417 | -24.333 |
| São Paulo | Bauru | -49.083 | -22.317 |
| São Paulo | Bocaina | -44.633 | -22.700 |
| São Paulo | Campos do Jordão | -45.591 | -22.739 |
| São Paulo | São Paulo | -46.650 | -23.417 |
| São Paulo | Carapicuíba | -46.836 | -23.865 |
| São Paulo | Cotia | -46.736 | -23.604 |
| São Paulo | Franca | -47.401 | -20.539 |
| São Paulo | Iguape | -47.555 | -24.708 |
| São Paulo | Itararé | -49.341 | -24.115 |
| São Paulo | Iporanga | -48.593 | -24.586 |
| São Paulo | Mogi das Cruzes | -46.188 | -23.523 |
| São Paulo | Juquiá | -47.635 | -24.321 |
| São Paulo | Ituverava | -47.781 | -20.506 |
| São Paulo | Itaporanga | -49.490 | -23.708 |
| São Paulo | Piquete | -45.176 | -22.614 |
| São Paulo | Santo André | -46.059 | -23.803 |
| São Paulo | Piracicaba | -47.649 | -22.725 |
| São Paulo | Juquiá | -47.635 | -24.321 |
| São Paulo | São José dos Campos | -45.974 | -22.901 |
| São Paulo | São Roque | -47.135 | -23.529 |
| São Paulo | Serra Negra | -46.701 | -22.612 |
| São Paulo | São Miguel Arcanjo | -47.991 | -24.067 |
| São Paulo | Américo Brasiliense | -48.100 | -21.720 |
| São Paulo | Campinas | -47.060 | -22.900 |
| São Paulo | Jundiaí | -46.924 | -23.232 |
| São Paulo | Ilha do Cardoso, São Paulo | -47.966 | -25.132 |
| São Paulo | Cananéia | -47.964 | -24.972 |
| Sergipe | Cristinápolis | -37.757 | -11.466 |
