# Supplementary material for: Niche Suitability Affects Development: Skull Asymmetry Increases in Less Suitable Areas
Source: PLoS One. 2015 Apr 15;10(4):e0122412. doi: 10.1371/journal.pone.0122412 (PMC4398368; doi:10.1371/journal.pone.0122412)
Supplement: S2 Appendix — List of museum specimens (N = 380) containing a description of the locality with geographic coordinates in longitude and latitude degrees, the number of individuals by locality, and the museum collection numbers of specimens of Akodon cursor used in this study. (DOCX) [file pone.0122412.s002.docx]

**Appendix S2.** **List of museum specimens**. List of museum specimens (N= 380) containing a description of the locality with geographic coordinates in longitude and latitude degrees, the number of individuals by locality, and the museum collection numbers of specimens of *Akodon cursor* used in this study.

Museu Nacional da Universidade Federal do Rio de Janeiro (MN). Garanhuns, Pernambuco (57 specimens) (-36.49; -8.88): MN - 18928, 18930, 18931, 18933, 18934, 18935, 18936, 18938, 19008, 19009 , 19010, 19013, 19014, 19015, 19016, 19017, 19018, 19019, 19020, 19021, 19022, 19023, 19024, 19026, 19027, 19028, 19030, 19031, 19032, 19033, 19034, 19035, 19036 , 19038, 19039, 19040, 19041, 19042, 19043, 19044, 19045, 19046, 19047, 19048 , 19049, 19114, 19115, 19116, 19118, 19121, 19122, 19123, 19124, 19125 , 19126, 19127, 19128. Viçosa, Alagoas (22 specimens) (-36.24; -9.36): MN – 12944, 13163, 13169, 13175, 13192, 13218, 13228, 18845, 18844, 18846, 18847, 18848, 18849, 18851, 18865, 19055, 19058, 12882, 12887, 12891, 12893, 12894. Feira, Bahia (32 specimens) (-38.97; -12.26): MN – 18901, 18902, 18903, 18904, 18905, 18906 , 18908, 18909, 13124, 18911, 18912, 18913, 18914 , 18915, 18916, 18917, 18918, 18919, 18952, 18953, 18954, 18966, 18967, 18968 , 18969, 18970, 18971, 18972, 19141, 19142, 19143, 29429. Ilhéus, Bahia (39 specimens) (-39.07; -14.79): MN - 8867, 8873, 8876, 8887, 8890, 8891, 8895, 8903, 8926, 8941, 8943, 8946, 8953, 8976, 8982, 8983, 8984, 8989, 8990, 8991, 8993, 8994, 8997, 9000, 9011, 9013, 9014, 9028, 9031, 9036, 9039, 9040, 9041, 9042, 9043, 9046, 9047, 9048, 9056. Além Paraíba, Minas Gerais (19 specimens) (-42.69; -21.87): MN – 5383, 5401, 7325, 7344, 7348, 7384, 7391, 7392, 7394, 7396, 7397, 7456, 7458, 7464, 7476, 7478, 7482, 7483, 7486. Teresópolis, Rio de Janeiro (19 specimens) (-42.95; -22.41): MN – 7106, 7107, 7113, 7114, 7115, 7116, 7117, 7109, 7118, 7119, 7121, 7122, 7123, 7124, 7128, 7130, 7132, 7135, 7136. Angra dos Reis, Rio de Janeiro (17 specimens) (-44.47; -22.99): MN – 61777, 61778, 61779, 61780, 61781, 61782, 72053, 72065 , 72051, 59114, 72071, 72089, 72114, 72115, 72138, 72139, 72101. Passos, Minas Gerais (27 specimens) (-46.60; -20.71): MN – 12790, 12792, 12793, 12797, 12802, 12805, 12810, 12812, 12813, 12814, 12815, 12816, 12817, 12818, 12825, 12827 , 12828, 12830, 12833, 12835, 12836, 12837, 12838, 12840, 12842, 12845 , 12846. Bom Conselho, Pernambuco (6 specimens) (-36.68; -9.17): MN – 18929, 18937, 18939, 19011, 19012, 19117. Capela, Alagoas (4 specimens) (-36.08; -9.41): MN – 12932, 12933, 12934, 12935. Caruaru, Pernambuco (9 specimens) (-35.95; -8.27): MN – 2264, 12964, 12956, 18853, 18854, 18855, 18856, 2266, 2263. Cristianápolis, Sergipe (3 specimens) (-37.75; -11.46): MN – 30598, 43904, 43905. Guararema, São Paulo (3 specimens) (-46.03; -23.40): MN – 24186, 24189, 24192. Quebrângulo, Alagoas (7 specimens) (-36.46; -9.32): MN – 18839, 18843, 18857, 18858, 18859, 18860, 18861. Santa Teresa, Espirito Santo (7 specimens) (-40.55; -19.88): MN – 5259, 5266, 5275, 5319, 5328, 5347, 5359. São Miguel dos Campos, Alagoas (8 specimens) (-36.09; -9.78): MN – 30599, 30601, 30602, 30603, 30604, 30605, 30606, 30607. Ubatuba, São Paulo (5 specimens) (-45.1; -23.41): MN - 5267, 5268, 5311, 5355, 5369.

Museu de Zoologia da Universidade de São Paulo (MZUSP). Salesópolis, São Paulo (21 specimens) (-45.9; -23.65): MZUSP – 10869, 10881, 10931, 10948,10864, 24261, 10955, 20947, 10853, 10823, 10769, 10772, 10854, 10878, 10826, 10856, 10973, 20949, 10843, 10936,10922. Salto de Pirapora, São Paulo (18 specimens) (-47.57; -23.65): MZUSP – 24527, 24550, 24413, 24610, 24531, 24522, 24563, 24701, 24576, 24526, 24561, 24420, 24534, 24556 , 24530, 24521, 24591, 24542. Iguapé, São Paulo (29 specimens) (-47.55; -24.70): MZUSP – 10681, 24175, 24169, 24174, 11450, 26785, 24178, 11417, 26782, 11421, 26797, 26798, 26780, 27410, 26774, 26784, 26783, 26796, 26781, 22802, 24985, 24981, 24983, 20632, 20635, 20636, 20637, 20638, 20630. Capão Bonito, São Paulo (24 specimens) (-48.41; -24.33): MZUSP – 29239, 29240, 29241, 29242, 29243, 29244, 29245, 29246, 29218, 29219, 29228, 29229, 29230, 29231, 29232, 29233, 29234 , 29235, 29237, 29238, 27203, 27198, 27204, 27214. Ilha do Cardoso, São Paulo (4 specimens) (-47.96; -25.13): MZUSP – 28388, 28379, 27753, 27754.
