# Supplementary material for: Niche Suitability Affects Development: Skull Asymmetry Increases in Less Suitable Areas
Source: PLoS One. 2015 Apr 15;10(4):e0122412. doi: 10.1371/journal.pone.0122412 (PMC4398368; doi:10.1371/journal.pone.0122412)
Supplement: S3 Appendix — (DOCX) [file pone.0122412.s003.docx]

**Appendix S3. Description of the 40 landmarks digitized on the ventral skull of the 380 specimens of *Akodon cursor*.**

Landmarks were defined as: 1, anteriormost point of the suture between nasals; 2-3, outermost point of the alveolus of the incisor; 4-5, anteriormost point of the incisive foramen; 6-7, posteriormost point of the incisive foramen; 8-9, rostral end of the zygomatic plate; 10-16, anteriormost point of the molar row; 11-17, contact point between the first and second molar tooth; 12-19, contact point between the second and third molar tooth; 13-19, posteriormost point of the molar row; 14-20, lateral paracone of the first molar; 15-21, medial paracone of the first molar; 22, posteriormost point of the suture between palatines and the anterior border of the mesopterygoid fossa; 23-24, posteriormost point of the orbit; 25-27, anteriormost point of the eustachian tube; 26-28, suture between the basisphenoid and basioccipital at the contact with the auditory bulla; 29-30, anteriormost border of the paramastoid process; 31-33, anteriormost external border of the ectotympanic; 32-34, posteriormost border of the masseteric tubercle; 35-36, lateralmost point of the occipital condyle; 37, anteriormost point of the foramen magnum; 38, posteriormost point of the foramen magnum; 39-40, anteriormost border of the occipital condyle.
